# Distortion-product Otoacoustic Emissions in Diagnostic Versus Portable Equipment: A Comparison of Animal Models

**DOI:** 10.1055/s-0044-1801314

**Published:** 2025-05-07

**Authors:** Gabriela Guenther Ribeiro Novanta, Vanessa Silva Pinto, Juliana Gusmão de Araújo, Lucieny Martins Serra, Andre Luiz Lopes Sampaio

**Affiliations:** 1 Laboratory for Teaching and Research in Otorhinolaryngology, School of Medicine, University of Brasília, Brasília, DF, Brazil

**Keywords:** otoacoustic emissions, hearing, cochlea, rats

## Abstract

**Introduction** Many protocols carried out in animal studies use equipment developed for humans. Therefore, the equipment available on the market must be known in detail, as well as how the criteria to be evaluated are presented.

**Objective** To analyze the existence of an association between the amplitude and signal-to-noise ratios of distortion-product otoacoustic emissions using two methodologies (diagnostic and portable/screening equipment) in animal models.

**Methods** Experimental study approved by the Animal Use Ethics Committee, with a sample of 28 female Wistar rats, which were subjected to anesthesia, manual otoscopy, and distortion-product otoacoustic emission (DPOAE) examination at 4 to 8 kHz with the 2 pieces of equipment.

**Results** The mean amplitude values with the ILO (Otodynamics Ltd., Hatfield, United Kingdom) and OtoRead equipment (Interacoustics A/S, Middelfart, Denmark) were respectively 20.5 dB and 7.1 dB at 4 kHz; 31.8 dB and 19.37 dB at 6 kHz; and 31.4 dB and 25.1 dB at 8 kHz. The mean signal-to-noise ratios with the ILO and OtoRead equipment were respectively 20.9 dB and 25.1 dB at 4 kHz; 35.8 dB and 37.0 dB at 6 kHz; and 39.7 dB and 40.6 dB at 8 kHz. There was no statistically significant difference in signal-to-noise ratios at 6 and 8 kHz. When the data were classified as normal/abnormal, 100% agreement was found between the methodologies.

**Conclusion** An association was found in the analysis of the mean signal-to-noise ratio at 6 and 8 kHz between the 2 methodologies (diagnosis and portable/screening equipment).

## Introduction

Animal models have been useful and part of biomedical experimental research for centuries. 1 2 Since 1980, the use of rats for hearing research purposes has increased, especially in structural and functional studies. 3

Rodents have low maintenance costs and can be easily kept in vivaria, and their structural similarities with the human ear raise interest in experimental research. 4 5 6 The organization of their cochlea is comparable to that found in mammals and humans, although their hearing encompasses a wider range of frequencies, from 0.2 to 90 kHz, whereas in humans it ranges from 0.02 to 20 kHz. ^2^

Various techniques are used to evaluate hearing in animal models. In 1979, Francis 7 described the Preyer reflex as useful for evaluating rats' sensitivity to volume, despite not being a good predictor of the conditioned-response audiogram. More recent rodent studies performed histological analyses 8 9 10 and assessed otoacoustic emissions (OAEs) 9 11 12 13 14 and auditory brainstem response (ABR) 9 10 15 to better understand topics related to noise exposure, 9 13 otoprotection, 11 12 13 14 ototoxicity, 10 11 14 presbycusis, 12 tinnitus, 8 hyperbilirubinemia, 15 and other hearing conditions.

Otoacoustic emissions are low-intensity sounds produced in the cochlear outer hair cells and propagated toward the middle ear and external auditory meatus. They can be detected in the ear canal with a sensitive microphone 16 17 and are classified according to the occurrence of external stimulation. Distortion product otoacoustic emissions (DPOAEs) are often used in experimental studies, in part because of their relative frequency specificity. They are generated by 2 pure tones ( *f1* and *f2* ) presented concomitantly, with a preferable distance between them at the *f1* / *f2* ratio of 1.22 to generate an ideal distortion product with greater amplitude in the human cochlea. 18 19

The scientific literature evaluates a wide range of frequencies—some studies analyze up to 8 kHz, 20 9 kHz, 11 21 12 kHz, 22 23 24 and 22.6 kHz. 25 As the rats' hearing reaches higher frequencies, ranging from 0.2 to 90 kHz, this evaluation method is interesting because it provides information specific to high frequencies.

Freitas et al. 26 carried out a study in 2009 to evaluate the sensitivity of DPOAE sand ABR in detecting ototoxicity secondary to different doses and forms of administration of cisplatin in rats. They concluded that ABR was more sensitive than DPOAEs. The study used only amplitudes at 3, 4, 6, and 8 kHz to evaluate DPOAEs and click stimuli at 0 to 3 kHz to perform ABR. Despite stating that ABR would be more sensitive than OAEs, the authors make clear the controversy on how to determine the ABR electrophysiological threshold in rats. Otoacoustic emissions are still the best option to study cochlear hair cells, and the protocols must be well defined, as complete as possible, and adapted to the anatomy of the animal studied.

Some of the protocols carried out in studies in animal models use equipment developed for humans. 11 12 22 27 Therefore, the equipment available on the market must be known in detail, as well as how the acquisition and analysis methodology is presented. Some studies in animals have established only the response amplitude as a criterion to interpret the examination, 9 16 28 or only the signal-to-noise ratio (SNR), 14 29 30 while others analyzed both criteria (amplitude and SNR). 11 12 13 Normal SNR classification values were found to be from 3 to 6 dB SPL, 13 14 16 although some studies chose to analyze only the variation of amplitude and SNR over time in the tests. 9 11

Protocols for acquiring and evaluating DPOAE on portable equipment are available, which are widely used for neonatal hearing screening and audiological diagnoses in clinical practice. Although few studies do so, issues related to commercial equipment must be addressed. Much research is carried out with equipment built specifically for animal study, hindering their replication in many study centers due to the lack of this level of technology.

The possibility of translating results into the human species is not yet clearly known. Therefore, this type of study must be conducted to verify whether it is feasible to use commercial devices in experimental animal research. The authors of some published studies have used models developed for humans and found that these devices efficiently evaluated cochlear integrity in animals. 12 22 The advantage of using portable screening equipment is the convenience of transportation, handling of the device, and fast response acquisition, which is very important and safe considering the anesthesia procedures.

In addition to screening equipment, there is also diagnostic equipment, which takes longer to collect results and is widely used for animal research ^14,29,30^ . This includes ILO 292 (Otodynamics Ltd., Hatfield, United Kingdom), which is considered the gold standard. 11

Therefore, the present study aimed to analyze two DPOAE acquisition methods to verify whether signal amplitude and SNR values and the pass/fail criterion were associated in Wistar rats, using a portable equipment protocol and diagnostic equipment, corroborating the applicability in experimental models and future translations to humans derived from these research works.

## Methods

This experimental study was developed at the Laboratory for Otorhinolaryngology Teaching and Research at University of Brasília (UnB), based on a multidisciplinary project approved by the Animal Use Ethics Committee, under UnB DoC protocol no. 7117/2015.

The sample comprised 45 female Wistar rats from the vivarium of Universidade Federal de Goiás, aged 2 months and 15 days, with an average weight of 190 g. The animals were kept in the vivarium under normal lighting conditions, with free access to food and water, in natural sleep-wake cycles, kept in boxes, divided into groups, and handled according to the standards recommended by Colégio Brasileiro de Experimentação Animal (COBEA).

The animals were initially weighed to calculate drugs administration doses for anesthesia as follows: ketamine hydrochloride (65 mg/kg [50 mg/mL]) and xylazine (6.5 mg/kg [20 mg/mL]).

Before the DPOAE test, the animals were submitted to otoscopy by an otorhinolaryngologist researcher to evaluate the external auditory canal and tympanic membrane. Animals with abnormal otoscopy would be excluded from the study.

Two pieces of equipment with the same configuration were used for the tests, as follows: analyses at 4 to 8 kHz and stimuli with 2 pure tones ( *f1* and *f2* ), at an *f1/f2* frequency ratio equal to 1.22, and a stimulus intensity fixed at 70 dB SPL. There was a total of 1,000 acquisitions for the ILO equipment and a total of acquisitions lasting 4 seconds per frequency tested on the OtoRead equipment.

The research used the portable OtoRead OAE equipment (Interacoustics A/S, Middelfart, Denmark), connected to the equipment's printer, and the ILO 292 Echoport system evoked OAE diagnostic equipment with the ILO V6 software (Otodynamics Ltd.), connected to a computer.

Adapted probes were used in the external auditory canal for both pieces of equipment in order to avoid discomfort to the animals. The adaptation was performed with an olive used for neonates and approved for use after calibration in the test cavity. Distortion product otoacoustic emissions were evaluated in all animals in the following order: equipment 1 (portable), DPOAE in the left ear, and then in the right ear. The same analysis was then carried out with equipment 2 (diagnosis). Two records were made on each piece of equipment with an interval of 1 min between collections and the mean value was used as a result.

The responses used in the descriptive analysis were the means, minimum, and maximum values, and standard deviations (SD) of amplitude and SNR. Distortion product otoacoustic emissions were considered present in both pieces of equipment when the amplitude values were ≥ −5 dB SPL and the SNR values were ≥ 6 dBSPL. 31 32

Inductive statistical analysis was performed with the paired samples t-test to compare the mean amplitudes and SNR (continuous variables) with the Spearman correlation (the Shapiro-Wilk test resulted for non-parametric data) to verify the trend of change in continuous variables using both devices, though not necessarily at a constant rate. Lastly, the McNemar test was used to compare nominal variables, that is, OAE classification as either present or absent at 4 to 8 kHz, in both pieces of equipment. The program used was Jamovi Software, version 1.2.25 Jamovi, Sydney, Australia), with the significance set at *p* < 0.05.

## Results

The sample consisted of 28 animals that had normal otoscopy according to the established inclusion criteria. Due to wax in the external meatus or opacity in the tympanic membrane, 17 animals were excluded.

The ears were grouped in the 2 tests, as the analysis found no difference between the right and left ears (paired samples t-test – all *p* > 0.05). Thus, the analysis sample consisted of 56 ears.

The minimum and maximum values, standard deviation (SD), and *p* -values found with the paired samples t-test for signal amplitude and SNR at 4, 6, and 8 kHz are respectively shown in Tables 1 and 2 , according to the methodology used to acquire DPOAEs (diagnostic ILO and portable OtoRead). There is a statistically significant difference of the means between the 2 pieces of equipment, in the frequencies of 4, 6, and 8 kHz in the amplitude criterion and SNR means at 4 kHz.

**Table 1:** Mean, standard deviation, and minimum and maximum amplitude of the distortion-product otoacoustic emissions of the 56 ears per methodology (diagnostic ILO and portable OtoRead)

Frequencies	ILO				OtoRead				*p*
	Mean	Min	Max	SD	Mean	Min	Max	SD	
**4 kHz**	20.5	7	30	5.01	7.1	0	15	4.10	< 0.001*
**6 kHz**	31.8	12.1	39.6	5.22	19.3	12	28	3.98	< 0.001*
**8 kHz**	31.4	12.5	37.8	4.51	25.1	11	33	5.38	< 0.001*

**Abbreviations:** max, maximum; min, minimum; SD, standard deviation.

**Note:** *Statistical paired-samples *t* -test; * *p* < 0.05.

The mean DPOAE amplitude values are higher with ILO at the evaluated frequencies, with a statistically significant difference at all frequencies ( *p* < 0.05).

**Table 2:** Mean, standard deviation, and minimum and maximum signal-to-noise ratio of the distortion-product otoacoustic emissions of the 56 ears per methodology (diagnostic ILO and portable OtoRead)

Frequencies	ILO				OtoRead				*p*
	Mean	Min	Max	SD	Mean	Min	Max	SD	
**4 kHz**	20.9	10	30.8	5.29	25.1	12	35	5.99	< 0.001*
**6 kHz**	35.8	15.3	45.5	5.65	37.0	24	46	5.66	**0.185**
**8 kHz**	39.7	28.5	48.4	4.75	40.6	29	51	5.57	**0.238**

**Abbreviations:** max, maximum; min, minimum; SD, standard deviation.

**Note:** *Statistical paired-samples *t* -test; * *p* < 0.05.

The SNR analysis shows higher mean values with OtoRead, and it was statistically significant only at 4 kHz. No significant differences were found at the other frequencies tested. These data demonstrate that the means of SNR are highly associated with the 2 acquisition methods at 6 and 8 kHz.

The assessment of the dependence between the variables with Spearman's correlation regarding amplitude ( Table 3 ) and SNR ( Table 4 ) for all frequencies among the method of acquisition of DPOAE was positive—that is to say, the increase in one variable in one method corresponds to the increase in the other variable in the other method and the *p* -value (was statistically significant at all frequencies), demonstrating an association between amplitude and SNR between DPOAE acquisition methods.

**Table 3:** Correlation between methodologies (diagnostic ILO and portable OtoRead), considering the amplitude of the DPOEs in the 56 ears

Amplitude	4 kHz (ILO)	6 kHz (ILO)	8 kHz (ILO)
**4 kHz (OtoRead)**	r = 0.493 *p* ≤ 0.001		
**6 kHz (OtoRead)**		r = 0.383 *p* = 0.004 ^*^	
**8 kHz (OtoRead)**			r = 0.494 *p* ≤ 0.001

**Abbreviation:** DPOEs, distortion product otoacoustic emissions.

**Note:** Spearman's correlation statistical test; * *p* < 0.05.

**Table 4:** Correlation between methodologies (diagnostic ILO and portable OtoRead), considering the signal-to-noise ratio of the DPOEs in the 56 ears

Signal-to-noise ratio	4 kHz (ILO)	6 kHz (ILO)	8 kHz (ILO)
**4 kHz (OtoRead)**	r = 0.407 *p* = 0.002 ^*^		
**6 kHz (OtoRead)**		r = 0.276 *p* = 0.040 ^*^	
**8 kHz (OtoRead)**			r = 0.323 *p* = 0.015 ^*^

**Abbreviation:** DPOEs, distortion product otoacoustic emissions.

**Notes:** Spearman's correlation statistical test; * *p* < 0.05.

The association analysis between the results of the two DPOAE acquisition methods according to the pass/fail criterion found no statistically significant differences between the two devices at the frequencies studied ( *p* > 0.05) ( Table 5 ).

**Table 5:** Association of amplitude and SNR results of the DPOEs according to the pass/fail criterion per methodology (diagnostic ILO and portable OtoRead)

Frequencies	ILO		OtoRead		
	Present	Absent	Present	Absent	*p*
**Amplitude:** ** 4 kHz**	56	0	56	0	NS
**SNR:** ** 4 kHz**	56	0	56	0	NS
**Amplitude:** ** 6 kHz**	56	0	56	0	NS
**SNR:** ** 6 kHz**	56	0	56	0	NS
**Amplitude:** ** 8 kHz**	56	0	56	0	NS
**SNR:** ** 8 kHz**	56	0	56	0	NS

**Abbreviations:** DPOEs, distortion product otoacoustic emissions; NS, not significant; SNR, signal-to-noise ratio.

**Note:** McNemar test; *p* < 0.05.

## Discussion

The present study found no significant differences in the laterality of responses in these animals. Other studies in rodents likewise did not demonstrate asymmetries between the ears. 22 30 Hence, the ears were grouped.

The way to interpret these results has not yet been standardized. Some studies evaluated only the response amplitude 12 21 23 33 or only the SNR, 24 34 whereas other ones evaluated both results. 11 The SNR is widely used to assess animals and is described by Ceylan et al. (2019) 13 as more reliable than only the response amplitude. The present study used both variables because it aimed to validate the DPOAE acquisition methods for practice in experimental rodent studies.

The DPOAE acquisition protocols used in the current research was chosen according to analyses of previous studies. In research that aimed to verify the effect of pomegranate extract in reducing cisplatin-induced ototoxicity, 35 the researchers performed DPOAE examinations in Wistar rats using the ILO V6 software. Distortion product otoacoustic emissions results at 4, 6, and 8 kHz were equivalent to those in the present research. Another study, 26 likewise in Wistar rats, used the same protocol as the one used in the present research, with 1,000 acquisitions, the same *f1* / *f2* intensity (70 dB), and considering SNR results of at least 6 dB SPL. However, in comparison with other studies, many of them used the minimum SNR result of 3 dB SPL. 14 21 28 29 Most studies researched 12 13 21 23 24 33 used an *f1* / *f2* ratio of 1.22, but with stimuli fixed at 65 and 55 dB, though one study 20 fixed it at 55 dB. These data demonstrate that there is no single protocol yet to perform DPOAE, although there is a protocol outline.

Studies using another diagnostic equipment (Madsen Capella ^2^ , GN Otometrics, Taastrup, Denmark) identified an increase in SNR at higher frequencies, from 2 kHz onwards, 36 and low values at 0.996 and 1 kHz. 37 This trend of increased response with increasing frequency (higher frequencies) is justified by the fact that the most intense noise levels occur at lower frequencies and is due to the proportions of the head and external auditory meatus of rats, which provide DPOAE responses below 2 kHz equivalent to those of noise. 38 For this reason, it was decided to analyze the responses only at 4 kHz onwards as, at this frequency, DPOAEs begin to increase significantly compared to lower frequencies.

The study by Salihoglu et al. (2017), 39 with 20 Wistar rats and using the Madsen Capella ^2^ equipment, obtained a mean of amplitude 23 dB at 4 kHz, while in the present one, the mean was 20.5 dB with ILO and 25.1 dB with OtoRead. The values found at 6 kHz were lower than those found in the present research, with a mean of 32 dB with Madsen Capella ^2^ , 35.8 dB with ILO, and 37 dB with OtoRead. Lastly, values at 8 kHz were the least divergent, as the cited study found a mean of 38 dB with Madsen Capella ^2^ , and the present one found means of 39.7 dB with ILO and 40.6 dB with OtoRead.

Most published studies 11 21 24 34 used high-standard and high-cost diagnostic equipment, such as ILO, Audera, and Capella. More recent studies 12 27 used portable equipment, which assesses high frequencies with the same reliability and safety. One of the studies 12 aimed to evaluate the effect of melatonin in preventing cochlear hair cell dysfunction in mice. The authors evaluated DPOAEs at 6, 8, 10, and 12 kHz monthly, for 10 months, with the ERO-SCAN-MAICO Diagnostics equipment (MAICO Diagnostics GmbH, Berlin, Germany), a portable device similar to the one used in the present study. The mean values at 6 and 8 kHz were similar to those in the present study—although the animals used (Wistar rats and C57BL/6J mice) have different anatomical characteristics.

The present study found significant numerical differences in amplitude at the 3 frequencies analyzed (4, 6, and 8 kHz). No similar studies were found that could justify these differences. However, it is believed to be due to the equipment, its filters, and factory settings that cannot be modified that might consist of difference in the functioning between diagnostic and portable equipment. Even though higher individual values of response amplitude, a statistical positive association was found between the equipment analyzed, that is to say when the amplitude increases in diagnostic method, it also gets higher in portage technology.

This effect was not observed in the SNR, whose results at 6 and 8 kHz showed no numerical difference between the diagnostic and portable screening equipment. Even though the mean SNR values were slightly higher with ILO, indicating a difference between the devices, the Spearman correlation test found a dependence between the variables, in that the increase in one variable corresponds to the increase in the other one. Thus, the DPOAE acquisition methods are associated concerning the amplitude and SNR—which advises against data interpretation based only on the numbers found, since the examination results were concordant in 100% of the animals evaluated with both pieces of equipment according to pass/fail criteria.

Ceylan et al. (2019) 13 suggest that SNR is more reliable for analyzing DPOAE responses, as it is acquired with less variation in the animal model. This was also verified in the present study, as both devices had associated results and, especially, no numerical statistical distribution difference.

As for models other than experimental studies, Ciorba et al. (2008) 40 conducted a project to identify neonatal hearing loss and define early intervention strategies. They produced a report with data on otoacoustic emissions in healthy newborns and other ones who stayed at a neonatal intensive care unit (NICU). Three screening devices were used in the study, including the OtoRead and ILO 292 diagnostic equipment. They performed the transient evoked otoacoustic emissions test (TEOAE), and the results in healthy newborns between the devices were quite similar, with no significant differences in the pass/fail criterion. In the order of best performance, the OtoRead portable equipment was ahead of the ILO diagnostic equipment, in both healthy and NICU newborns. Statistically significant differences were found between SNR values obtained with diagnostic and portable equipment (OtoRead) at 3 and 4 kHz. The article also highlights that ILO 292 responses seem to improve high-frequency TEOAE components compared with data recorded with portable equipment. The results of this study (using TEOAE) are similar to those in the present one (using DPOAE), demonstrating the reliability of such results.

It should also be noted that when the data in this study was classified as normal/altered, using the same criteria for both devices, regardless of the methodology (diagnosis and portable/screening), the analysis of amplitude and SNR showed 100% agreement.

Given the results, performing DPOAE with portable/screening equipment in rodents appears to be a feasible possibility to assess their hearing when the objective is exclusively to monitor the status of the cochlea with a protocol whose examination parameters do not need to be modified. On the other hand, diagnostic equipment is recommended for studies that need to modify the type of stimuli and intensities and standardize protocols other than those provided by the manufacturer, since most screening devices do not allow this type of adjustment. Moreover, considering the preforming time of the exam by itself, the screening equipment takes no longer than the diagnostic one, and this might take into account in experimental condition under the use of anesthesia drugs.

The present study has some limitations, such as small sample size and the use of a protocol with few analyzed frequencies. Moreover, the sample did not include animals with mild hearing loss. This study was designed to demonstrate the reliability of using screening equipment, thus assisting researchers in future studies, as this research group found consistent and reproducible results 11 12 22 by using DPOAEs in Wistar models.

## Conclusion

An association was found between the 2 methodologies (diagnosis and portable/screening equipment) and a 100% correspondence was found in pass/fail protocol. The variability of the results in amplitude of response seems to be bigger than SNR once there was a numerical statistically significant difference among individual values between the diagnostic and portable devices but positively associated.

The SNR was less variable between the 2 technologies, especially at 6 and 8 kHz, and a 100% equivalence was also found among the methods of acquisitioning pass/fail protocol. There was also a positive association between the methods. Therefore, higher SNR frequencies seem to be less variable.

The present study demonstrated the reliability of using a low-cost methodology that is quick to perform and easy to transport and handle, with a portable/screening device in rodent animal models.
